# El síndrome de norte-sur, una complicación de la asistencia circulatoria con ECMO Venoarterial. Reporte de caso.

**DOI:** 10.47487/apcyccv.v4i3.300

**Published:** 2023-09-30

**Authors:** Emmanuel Adrián Lazcano-Diaz., Karla Sue América Hurtado Belizario, Daniel Riveros-Nina, Aldo Pérez-Manjarrez, Daniel Manzur-Sandoval, Luis Baeza-Herrera, Gustavo Rojas-Velasco

**Affiliations:** 1 Terapia Intensiva cardiovascular, Instituto Nacional de Cardiología «Ignacio Chávez», Ciudad de México, México. Terapia Intensiva cardiovascular Instituto Nacional de Cardiología «Ignacio Chávez» Ciudad de México México

**Keywords:** Síndrome de Marfan, ECMO Venoarterial, Choque Cardiogénico, Marfan Syndrome, ECMO, venoarterial, Shock, Cardiogenic

## Abstract

Presentamos el caso de un paciente masculino de 34 años con diagnóstico previo de síndrome de Marfan que ingresó por Insuficiencia aórtica aguda secundario a dilatación aneurismática de la aorta torácica ascendente. En el posoperatorio se documentó choque cardiogénico poscardiotomía por lo que se inició soporte circulatorio con ECMO venoarterial periférico, el cual desarrolló hipoxemia por neumonía bacteriana y datos compatibles con síndrome norte-sur. Presentamos una revisión, estrategias de canulación no convencional y una alternativa diagnóstica para esta entidad.

## Abstract (en)

We present the case of a 34-year-old male patient with a history of Marfan syndrome who was admitted to the emergency room for acute aortic regurgitation secondary to aneurysmal dilation of the ascending thoracic aorta. In the postoperative period, post-cardiotomy cardiogenic shock was documented, so circulatory support was initiated with peripheral Veno-Arterial ECMO, which developed hypoxemia due to bacterial pneumonia and data compatible with North-South syndrome. We present a review, non-conventional cannulation strategies and a diagnostic alternative for this entity.

## Introducción

El choque cardiogénico secundario a bajo gasto poscardiotomía (CCBGP) es una complicación poco frecuente que se desarrolla después de una intervención quirúrgica cardiaca. Algunas series lo mencionan en un 6% ^1^, en otras hasta un 10% ^2^. Entre las estrategias de soporte para CCBGP está el soporte circulatorio con ECMO venoarterial (*Extracorporeal Membrane Oxygenation*, por su siglas en inglés. ECMO V-A) ^1^^-^^3^. Dentro de las complicaciones más frecuentes asociadas a este dispositivo tenemos las vasculares y hemorrágicas, pero en algunos casos se describe el desarrollo de hipoxemia refractaria conocida como síndrome norte-sur ^4^, el cual tiene efectos deletéreos en el sistema nervioso central y cardiovascular. Se presenta un caso de síndrome norte-sur (SNS) en un paciente con CCBGP y se revisa las estrategias de diagnóstico y tratamiento en esta complicación. 

## Reporte de caso

Hombre de 34 años, con antecedente de desprendimiento de retina bilateral y cirugía de David (sustitución de raíz aórtica y resuspención de válvula aórtica) 12 años previos a este internamiento; acude a urgencias por referir disnea y angina. La exploración física con signos vitales dentro de parámetros normales; soplo diastólico concordante con insuficiencia aórtica y electrocardiograma de 12 derivaciones con datos de sobrecarga de volumen de cavidades izquierdas. El ecocardiograma transtorácico (ECOTT) evidenció dilatación del ventrículo izquierdo con fracción de expulsión (FE) de 60% e insuficiencia aórtica grave. Angiotomografía de aorta torácica con dilatación de hasta 76 mm, dilatación de la arteria coronaria derecha, sin evidencia de disección aórtica. Por lo anterior, fue valorado por el *Heart Team* local quienes decidieron realizar cirugía de Bentall y de Bono además de la reconstrucción de la arteria coronaria derecha.

**Figura 1:**
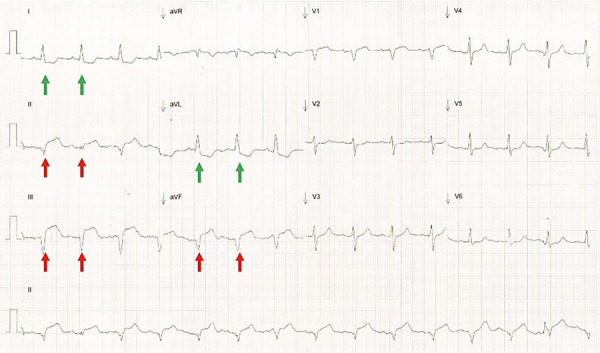
Electrocardiograma inmediato después de la cirugía. Electrocardiograma de 12 derivaciones al momento del ingreso a Terapia Intensiva Cardiovascular. Se evidencia elevación del segmento ST en derivaciones inferiores (flechas rojas) con cambios recíprocos en derivaciones laterales (flechas verdes).

A su ingreso a la Terapia Intensiva Cardiovascular después de la cirugía planeada, se identificó elevación del segmento ST en derivaciones DII, DIII y AVF **(**Figura 1**)**. El ecocardiograma transesofágico (ECOTE) evidenció acinesia en cara inferior, inferoseptal e inferolateral en sus tres tercios sugerentes de infarto de miocardio asociado a cirugía cardiaca (infarto tipo V). Por lo anterior, se realizó coronariografía diagnóstica urgente, evidenciándose adecuado flujo en arterias coronarias. Sin embargo, la evolución fue tórpida con incremento en el uso de inotrópicos y vasopresores sin mejoría en parámetros clínicos ni bioquímicos de perfusión tisular y se estableció el diagnóstico de CCBGP. Se realizó conexión de ECMO V-A periférico con descompresión ventricular con balón intraaórtico de contrapulsación (BIAC).

Tres días después del inicio del soporte circulatorio mecánico (SCM) se evidenció hipoxia en tronco con valores normales de oxigenación en extremidades inferiores **(**Tabla 1**)**. Se sospechó neumonía bacteriana que fue confirmada por una radiografía de tórax (presencia de consolidación basal izquierda), por lo cual se estableció el diagnóstico de síndrome norte-sur. Se ajustaron los parámetros ventilatorios sin resolución de la hipoxemia, por lo que se realizó una nueva configuración de ECMO V-A a ECMO venoarteriovenoso (ECMO V-AV); es decir, extracción de una vena y retorno a cavidades derechas del corazón y arterial aórtico. También se inició tratamiento antimicrobiano con base en meropenem y vancomicina.

**Tabla 1:** Valores de gasométricos del paciente al momento del diagnóstico de Síndrome Norte Sur

Sitio de muestra		PO_2_	PCO_2_	SaO_2_
ECMO	Premembrana	34 mmHg	50 mmHg	47%
	Posmembrana	227 mmHg	42 mmHg	100%
Extremidades inferiores	Arteria pedia derecha	201 mmHg	40 mmHg	100%
Extremidades superiores	Arteria radial derecha	51 mmHg	49 mmHg	78%
BIAC*	Aorta torácica descendente	52mmHg	49 mmHg	79%

* La gasometría del BIAC es equivalente a la obtenida de la arteria radial izquierda, debido a la posición anatómica del lumen distal de dicho dispositivo.

BIAC: balón intraaórtico de contrapulsación; ECMO: extracorporeal membrane oxygenation; mmHg: milímetros de mercurio; pCO_2_: presión parcial de dióxido de carbono; pO_2_: presión parcial de oxígeno; SaO_2_: saturación de oxígeno.

A las 48 h del inicio de la nueva configuración de soporte circulatorio y venoso, se documentó mejoría en la función hemodinámica y cardiovascular, por lo que a las 72 h del inicio de asistencia fue retirada sin complicaciones. El paciente fue egresado vivo a domicilio después de 25 días de estancia intrahospitalaria. 

## Discusión

Presentamos el caso de un paciente con CCBGP que requirió soporte circulatorio y respiratorio con una configuración no convencional debido a la complicación del SNS.

En la modalidad ECMO V-A periférico, la configuración tiene un sitio de mezcla del flujo proveniente del corazón y el retorno del dispositivo, el cual depende del inotropismo del músculo cardiaco y el flujo de retorno desde el ECMO. Si el gasto cardiaco del ventrículo izquierdo se encuentra normal, la mezcla será distal en comparación con un gasto cardiaco reducido en donde la mezcla puede ser a nivel del tronco braquiocefálico. El sitio de mezcla puede ser identificado mediante determinación de gases en sangre de arteria radial derecha e izquierda ^5^. 

**Figura 2:**
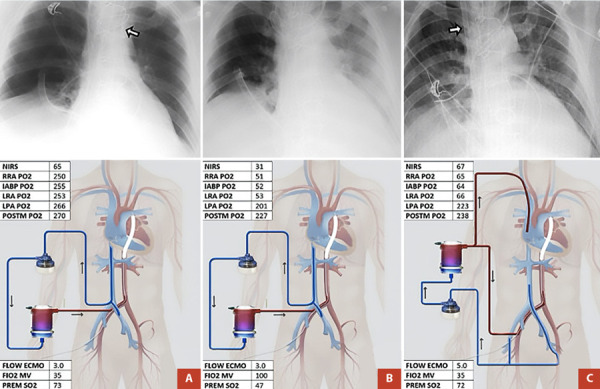
Síndrome de norte-sur. Transición de ECMO VA a ECMO V-AV. Correlación entre radiografía de tórax y hallazgos gasométricos. **Panel A.** Paciente en choque cardiogénico con asistencia circulatoria con ECMO V-A. La flecha indica la punta del balón de contrapulsación. **Panel B.** Misma configuración que el panel A; sin embargo, la radiografía de tórax muestra opacidad basal izquierda y evidencia de alteraciones gasométricas. **Panel C.** Configuración de ECMO V-AV para el tratamiento de las alteraciones gasométricas del panel B. La flecha indica la posición de la cánula de retorno en vena yugular derecha.

En condiciones normales, el flujo anterógrado desde el corazón tiene una concentración de oxígeno en valores normales (60-90 mmHg) y depende de la integridad del parénquima pulmonar para el adecuado intercambio de oxígeno, por lo que si este mecanismo se encuentra comprometido puede dar lugar a hipoxemia. Si la mezcla de oxígeno se encuentra distal a los troncos aórticos, existe riesgo de hipoxemia al corazón, cerebro y extremidades superiores ^5^^,^^6^**(**Figura 2**)**. Esto se conoce como el síndrome norte-sur o también llamado Síndrome de arlequín, debido a que las extremidades inferiores se mantienen con oxigenación por el ECMO dando lugar a una apariencia cianótica en las extremidades superiores e hiperémica en las inferiores.

ECMO V-A: ECMO venoarterial, ECMO V-AV: ECMO venoarteriovenoso. NIRS: Near-infrared spectroscopy, RRA: arteria radial derecha, PO2: presión parcial de oxígeno, IABP: balón de contrapulsación intraaórtico, LRA: arteria radial izquierda, LPA: arteria pedia izquierda, POSTM: post membrana, FLOW ECMO: flujo de ECMO, FiO2 MV: Fracción inspirada de oxígeno de ventilación mecánica, PREM SO2: Saturación de oxígeno pre membrana

El SNS es una complicación asociada al ECMO V-A en su modalidad periférica debido al flujo retrógrado (no evidenciándose en ECMO central debido al flujo anterógrado) ^7^^,^^8^. Entre las etiologías más frecuentemente descritas del síndrome norte-sur se encuentran el edema agudo pulmonar por dilatación del ventrículo izquierdo por la poscarga asociado al soporte con ECMO, y las infecciones pulmonares asociadas a la ventilación mecánica. La prevalencia del SNS varía entre un 8,8% ^9^^)^ hasta un 13,3% según los estudios recientes ^10^.

La descripción clásica del SNS fue hecha en neonatos en donde la inmadurez de los tegumentos permitía la identificación de los cambios de coloración en los tejidos. En nuestro caso, debido a la imposibilidad de obtener un acceso arterial en la extremidad superior izquierda (complejidad anatómica), optamos por obtener una muestra arterial del lumen distal del BIAC como análogo de la arteria radial izquierda y a través de esta se logró hacer el diagnóstico de SNS. La importancia del diagnóstico precoz del SNS radica en optimizar la recuperación miocárdica y evitar isquemia cerebral la cual resulta en déficit neurológico ^8^.

En la actualidad no se dispone de pautas claras en cuanto a su tratamiento. En este caso se optó por canulación a ECMO V-AV debido a que las maniobras convencionales (optimizar los parámetros del ventilador, aumentar el flujo del ECMO V-A junto con la descarga de ventrículo izquierdo) fallaron. Otras modalidades incluyen la conversión de ECMO V-A periférico a central, asistencia con doble bomba (en la que se utiliza una bomba adicional para dirigir el flujo de vuelta a la vena yugular) lo que parece reducir el riesgo de formación de coágulos, consumo de factores de coagulación y embolia pulmonar, etc. ^10^. 

En conclusión, la identificación oportuna de las complicaciones respiratorias asociadas al soporte circulatorio mecánico en pacientes con choque cardiogénico debe ser uno de los objetivos primordiales de los equipos dedicados a la atención de estos enfermos, con la finalidad de mejorar los desenlaces clínicos. 
